# No G6PD A- (G202A) variant detected among *Plasmodium falciparum*-positive patients in Awka, Southeast Nigeria: a hospital-based study

**DOI:** 10.5281/zenodo.18552048

**Published:** 2026-02-09

**Authors:** Moses Ikegbunam, Nwokike Uchechukwu, Harrison Abone, Ani Ezinne Grace, Mercy Ezeunala, Nnanna Joy, Nzeukwu Chibumma Immaculata, Joy Ogugua Igwe, Obiageli Okeke, Frances Nworji, Peter Ihekwereme

**Affiliations:** 1 Department of Pharmaceutical Microbiology and Biotechnology, Faculty of Pharmaceutical Science, Nnamdi Azikiwe University, Awka, Anambra State, Nigeria; 2 Molecular Research Foundation for students and scientists, Nnamdi Azikiwe University, Awka; 3 Department of Pharmacology and Toxicology, Nnamdi Azikiwe University, Awka, Nigeria; 4 Department of Parasitology and Entomology, Nnamdi Azikiwe University, Awka; 5 National Institute for Pharmaceutical Research and Development (NIPRD), Idu, Abuja, Nigeria; 6 Department of Biological Sciences (Microbiology Unit), Dennis Osadabay University, Asaba Delta State; 7 Department of Pharmaceutical microbiology and Biotechnology, Faculty of Pharmaceutical Science, Abia state University, Uturu, Abia State; 8 Department of Zoology, Nnamdi Azikiwe University, Awka, Nigeria; 9 Department of Applied Biochemistry, Nnamdi Azikiwe University, Awka, Nigeria

## Abstract

**Background:**

Glucose-6-phosphate dehydrogenase (G6PD) deficiency, prevalent in malaria-endemic regions, has been associated with a reduced risk of severe malaria due to impaired parasite growth in deficient erythrocytes. The G6PD gene, located on the X chromosome, harbours various mutations associated with differing enzyme activity levels. This study investigates the prevalence of G6PD deficiency variants and their impact on parasite density and haemoglobin levels among malaria-positive patients in Awka, Anambra State, Nigeria.

**Materials and Methods:**

Blood samples were collected from 100 malaria positive participants; 64 participants with complete genotyping and clinical data were included in the analysis and screened for the A376G and G202A variants using PCR and Sanger sequencing.

**Results:**

Molecular analysis indicated that the B variant (normal) was predominant, with 83% of the participants possessing this variant. None of the participants tested had the A- variant, associated with G6PD defciency, suggesting no evidence of the G202A (A−) variant in this hospital-based sample. The B variant and the A+ variant showed no significant impact on the haemoglobin and parasitaemia levels of the study participants.

**Conclusions:**

The findings support the absence of the G202A (A−) variant in this cohort and show no detectable differences in parasitaemia or haemoglobin between A+ and B genotypes. Broader genotyping and/or G6PD enzyme activity testing in community representative samples is recommended before drawing population-level conclusions or informing treatment policy.

## Introduction

Malaria is a major public health problem across many tropical and subtropical regions worldwide, with the highest burden in sub-Saharan Africa. Evolutionary pressure from malaria has shaped the frequency of host genetic polymorphisms affecting red blood cell biology, which may modify the risk of severe malaria infections [1]. One of the most common inherited factors is Glucose-6-phosphate dehydrogenase (G6PD) deficiency, characterised by reduced enzyme activity and an X-linked inheritance pattern [2,3]. The geographical distribution of G6PD deficiency overlaps with malaria endemicity, suggesting a possible selective advantage, although the underlying protective mechanisms remain incompletely understood [3,4].

G6PD deficiency confers both potential advantages and risks in the context of malaria. Individuals with G6PD deficiency have been reported to exhibit lower parasite densities and a reduced risk of severe malaria in some settings. However, defciency also predisposes to oxidative stress-induced haemolysis, triggering onset of acute haemolytic anaemia [5,6]. This is particularly relevant for 8-aminoquinoline antimalarials such as primaquine, used to target *Plasmodium vivax/ovale* hypnozoites and gametocytes, because these drugs can cause dose-dependent oxidative haemolysis in patients with G6PD deficiency [5,6].

The G6PD gene exhibits considerable allelic diversity, with over 200 variants associated with suboptimal enzyme activity [7]. In sub-Saharan Africa, three predominant variants B, A, and A-display varying levels of enzyme activity, with implications for malaria outcomes [5]. While the A-variant has been linked to protection against malaria, the extent of this protection varies across different studies [5]. Other endemic regions feature distinct G6PD variants, such as G6PD Mediterranean [1,2] and G6PD Mahidol [5,8], which have also been associated with malaria-related phenotypes.

This study elucidated the prevalence of G6PD genotypes in Awka, Anambra State, and explored their influence on malaria parasite density and haemoglobin levels among malaria-positive patients. Examining the relationship between genetic factors and malaria parameters will contribute to a deeper understanding of malaria pathogenesis and inform strategies for disease management in endemic regions. Importantly, because this is a hospital-based, malaria-positive cohort, the study evaluated associations within cases and does not directly estimate infection risk (‘susceptibility’) in the general population.

## Methodology

The study to determine the allelic diversity of G6PD genotypes and its effect on parasitaemia and haemoglobin concentration in malaria patients in Awka, Anambra State was carried out at the Chukwuemeka Odumegwu Ojukwu Teaching Hospital (COOUTH), Awka, Anambra State, between July 2019 and November 2020. Awka is a metropolis in Anambra State, Southeastern Nigeria with an estimated population of 244,005 [9].

### Study population

Blood samples were collected from 100 participants infected with malaria, as confirmed by RDT. Appropriate ethical approval and parental consent for children who participated in the study were obtained. Participants were randomly selected, with 36 males and 64 females participating in the study. Of these, 64 participants with complete genotyping and haemoglobin/parasitaemia data were included in the final analysis.

### Parasite counts

Parasite density was determined on Giemsastained thick blood films by a WHO-certified malaria microscopist immediately after sample collection. Thick films were prepared, air dried, and stained with 10% Giemsa for 10 min. Microscopy was performed using a light microscope with a 100× oil immersion objective. Asexual parasites were counted against 200 white blood cells (WBCs), and parasite density was estimated using an assumed WBC count. Parasite density (parasites/μl) was calculated as:

Parasites/*μ*l = (Number of parasites counted × 8,000) / 200

where 8,000 WBC/*μ*l was used as the assumed WBC count for all participants. Parasite density values were recorded as parasites/μl and used for subsequent analyses.

### Haemoglobin concentration

Haemoglobin concentration was measured at the point of blood collection using a HemoCue® photometer (HemoCue AB, Ängelholm, Sweden). Briefly, fresh whole blood was collected and a small volume was introduced into a HemoCue microcuvette according to the manufacturer’s instructions. The filled microcuvette was inserted into the photometer for photometric determination of haemoglobin concentration, and results were recorded immediately in g/dL. The device was operated with routine quality control in line with the manufacturer’s recommendations, including verification with the instrument’s built in self-check and/or control cuvettes where available.

### G6PD Genotyping and sequencing

Genomic DNA was extracted from blood samples using the Quick-DNA Miniprep Plus Kit (Zymo Research) from 200 μl of blood according to the manufacturer’s protocol. The polymorphic region of the G6PD gene was genotyped by polymerase chain reaction (PCR). In the PCR reaction, 3 μl of the extracted DNA was amplified in a 25 ul reaction consisting of 12.5 μl of One Taq Quick-Load 2X Master Mix (New England Biolabs Inc.), 0.5 μl of forward (5′-GCCCCTGTGACCTCCCGCCA-3′) and reverse (5′-GCAACGGCAAGCCTTACATCTGG-3′) primers, and 8.5 μl of nuclease-free water. The amplification conditions include an initial denaturation step at 94°C for 5 min, followed by 35 cycles of denaturation at 94°C for 45 seconds, primer annealing step at 65°C for 1 min, and strand extension at 72°C for 1 min. This was followed by a final extension at 72°C for 5 min. The PCR products were resolved on a 1.5% agarose gel stained with Ethidium Bromide. The results were viewed using a UV transilluminator.

Subsequently, the PCR products were purified using Exosap (Exo-SAP-IT, USB, Affymetrix, USA), according to the manufacturer’s protocol. The purified product was then used as a template for DNA sequencing by Eurofins MGW. G6PD polymorphisms were identified by comparing the sequencing results with the G6PD reference sequence gene (NG_009015.2) using the Codoncode Aligner 4.0 software (http://www.codoncode.com).

### Statistical analysis

All data were analysed using GraphPad Prism software, version 10.1.2. Data were grouped according to G6PD variation. Descriptive statistics were determined. The association of G6PD variation and parasitaemia or haemoglobin profiles, in males and females, respectively, was analysed using an unpaired t-test or Mann–Whitney U test, as appropriate based on data distribution. Statistical significance was considered at a p-value < 0.05. Given the small number of A+ males (n=2), genotype stratified comparisons in males should be interpreted as exploratory.

### Ethical clearance

The study was conducted in accordance with the Declaration of Helsinki and approved by the Institutional Review Board/Ethics Committee of Chukwuemeka Odimegwu Ojukwu Teaching Hospital, Awka, Nigeria (COOUTH/CMAC/ETH.C/Vol.1/0035).

## Results

**Figure 1:**
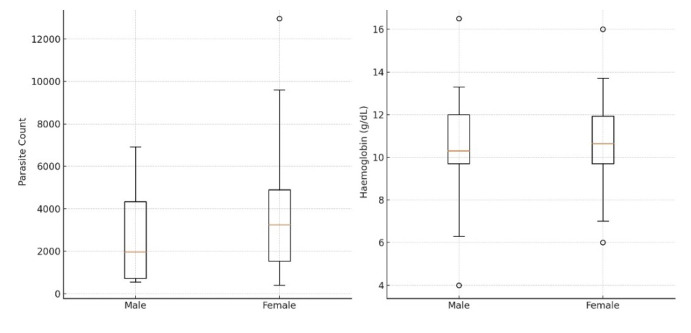
Parasitaemia (left) and haemoglobin levels (right) in study participants

Overall, 64 of the 100 malaria patients who enrolled in this study were included in the analysis. Female malaria patients constituted 67% (n=43) of the study population while 33% (n=21) of the study participants were male. The average (±SD) age of the female (range 1-44 yrs) and male (range 12-42 yrs) participants was 24.49 ± 9.22 and 22.95 ± 7.48, respectively. The parasitaemia and haemoglobin levels of study participants are shown in Figure 1. Participants included were those with complete genotyping and clinical data.

### Prevalence of G6PD variants

PCR and subsequent genome sequence analysis showed the presence of the B (376A/202G) and A+ (376G/202G) variants at the assayed loci (Table 1). The B variant was predominant in both females (79.07%) and males (90.48%). None of the participants tested carried the A− (202A) variant; all participants were 202G at the assayed position. Among females, genotypes at the A376G locus were called as either 376A/376A or 376G/376G; no heterozygous (376A/376G) calls were observed in this dataset.

**Table 1:** G6PD variants in study participants. N designates the normal polymorphism at position

	G6PD Variants			
Gender	B (376A)	A+ (376G)	N (202G)	A- (202A)
Male	19 (90.48%)	2 (9.52%)	2 1(100%)	0
Female	34 (79.07%)	9 (20.93%)	43(100%)	0

### Effect of G6PD genotype on parasitaemia levels in patients

**Figure 2:**
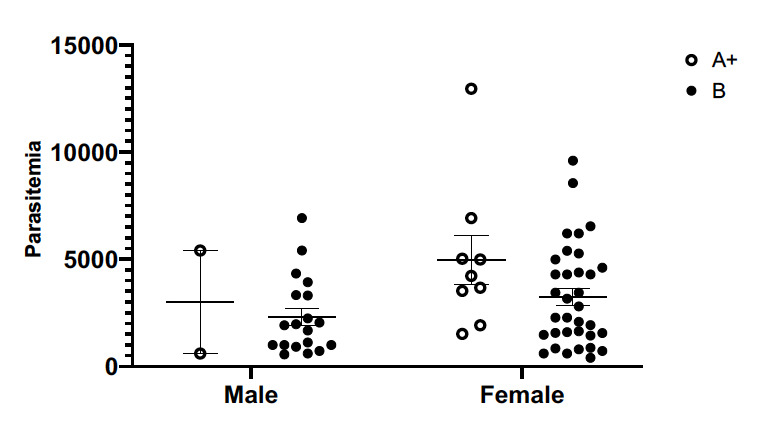
Scatter plot showing the relationship between G6PD genotype and parasitaemia.

G6PD A+ and B genotypes showed no significant impact on the parasitaemia profile of male and female subjects (Figure 2).

### Effect of G6PD genotype on haemoglobin level

**Figure 3:**
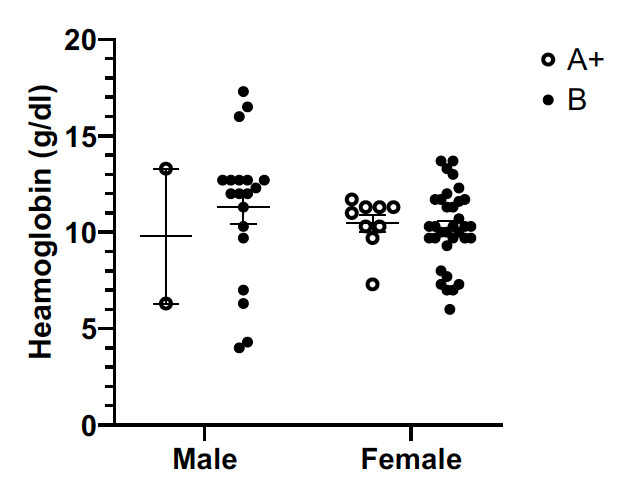
Scatter Plot showing relationship between G6PD genotype and haemoglobin.

Analysis of the haemoglobin profiles of malaria patients with either the G6PD B or A+ genotype (Figure 3) showed that there was no significant effect of G6PD genotypes (A+, B) on the haematological profiles both male (p = 0.95) and female (p = 0.64) subjects.

## Discussion

In this hospital-based cohort of malaria positive patients in Awka, we detected only the B (376A/ 202G) and A+ (376G/202G) variants at the assayed loci, and no participant carried the G202A (A-) allele. This observation should be interpreted as absence of G202A in this sample rather than an estimate of population prevalence.

Absence of the G202A allele among malaria patients has been reported in some African settings, including studies from Ethiopia [12,13] highlighting geographic heterogeneity in G6PD variants. In addition, because we genotyped only two loci (A376G and G202A), other deficiency associated alleles could be present but undetected; therefore, broader genotyping and/or phenotypic testing is required to characterise local G6PD deficiency comprehensively.

Because G6PD is X-linked, males are typically either normal or hemizygous deficient, whereas females may be homozygous or heterozygous. Random X-chromosome inactivation in heterozygous females results in red blood cell mosaicism and a wide range of measured enzyme activity. This biology reduces the sensitivity of qualitative point-of-care G6PD tests in females and increases the risk of misclassification around clinically relevant thresholds [14,23-25]. These considerations support WHO-recommended G6PD testing prior to primaquine based radical cure and underscore the value of combining genotyping with quantitative enzyme activity testing when evaluating local risk [3,14].

In this dataset, the mildly deficient A+ genotype was not associated with parasitaemia or haemoglobin compared with the B genotype. Interpretation of male subgroup comparisons is limited by the very small number of A+ males (n=2). The absence of heterozygous calls among females at the A376G locus is unusual for an X-linked trait and may reflect limited sample size, selection of malaria-positive hospital attendees, or technical/genotype-calling constraints; female genotype proportions are therefore interpreted cautiously. Prior studies have reported variable associations between G6PD genotypes and parasite density, with more consistent effects often observed for intermediate or severe deficiency variants rather than A+ [19,20].

Overall, our findings indicate that within this malaria-positive cohort, no G202A (A-) variant was detected and no association was observed between A+ and parasitaemia or haemoglobin. Larger community representative studies incorporating broader genotyping and quantitative G6PD enzyme activity testing are warranted to estimate population prevalence and to inform safe use of 8-aminoquinolines, which may precipitate acute haemolytic anaemia in G6PD-deficient individuals [3,5,6,15].

### Limitations

This was a hospital-based, malaria-positive cohort; therefore, the study assesses associations among cases and does not directly estimate malaria infection risk (‘susceptibility’) in the general population. Only two G6PD loci (A376G and G202A) were genotyped, which does not capture the full allelic diversity of G6PD variants in West Africa; absence of G202A in this cohort should not be interpreted as absence of G6PD deficiency overall. Enzyme activity was not measured, and genotype phenotype correspondence particularly in females because of X-chromosome inactivation cannot be inferred from these data alone. In addition, qualitative point-of-care tests can misclassify heterozygous females due to mosaicism. Finally, the small number of A+ males limited statistical power for subgroup comparisons, and the absence of heterozygous calls among females should be interpreted cautiously.

## Conclusions

In conclusion, among the malaria-positive patients analysed in this hospital-based study, no evidence of the G6PD G202A (A-) variant was detected in Awka, Anambra State. The A+ variant showed no detectable association with parasitaemia or haemoglobin when compared with the B variant in this dataset. Larger, community-representative studies that combine broader genotyping with G6PD enzyme activity testing are recommended before drawing population level conclusions or informing treatment policy where G6PD status is relevant.
